# Nursing Lecturers’ Perception and Experience of Teaching Cultural Competence: A European Qualitative Study

**DOI:** 10.3390/ijerph18031357

**Published:** 2021-02-02

**Authors:** Isabel Antón-Solanas, Isabel Huércanos-Esparza, Nadia Hamam-Alcober, Valérie Vanceulebroeck, Shana Dehaes, Indrani Kalkan, Nuran Kömürcü, Margarida Coelho, Teresa Coelho, Antonio Casa-Nova, Raul Cordeiro, Enrique Ramón-Arbués, Sergio Moreno-González, Elena Tambo-Lizalde

**Affiliations:** 1 Department of Physiatry and Nursing, Faculty of Health Sciences, Universidad de Zaragoza, Calle Domingo Miral s/n, 50009 Zaragoza, Spain; 2 Faculty of Health Sciences, Universidad San Jorge, Autovía Mudéjar, km. 299, Villanueva de Gállego, 50830 Zaragoza, Spain; eramon@usj.es (E.R.-A.); smoreno@usj.es (S.M.-G.); 3 Servicio Aragonés de Salud, Miguel Servet Women’s and Children’s Hospital, Paseo Isabel la Católica, 1-3, 50009 Zaragoza, Spain; hamamnadia@gmail.com; 4 Department of Nursing, AP University of Applied Sciences and Arts, Lange Nieuwstraat 101, 2000 Antwerpen, Belgium; valerie.vanceulebroeck@ap.be (V.V.); shana.dehaes@ap.be (S.D.); 5 Faculty of Health Sciences, Halit Aydin Campus 38, Istanbul Aydin University, Sefaköy-Küçükçekmece, Istanbul 34295, Turkey; indranikalkan@aydin.edu.tr (I.K.); nurankomurcu@aydin.edu.tr (N.K.); 6 Instituto Politécnico de Portalegre, School of Education and Social Science, Praça do Município 11, 7300-110 Portalegre, Portugal; margco@ipportalegre.pt (M.C.); teresa.coelho@ipportalegre.pt (T.C.); 7 Instituto Politécnico de Portalegre, School of Health Sciences, Praça do Município 11, 7300-110 Portalegre, Portugal; casanova@ipportalegre.pt (A.C.-N.); raulcordeiro@ipportalegre.pt (R.C.); 8 Instituto de Investigación Sanitaria, Hospital Universitario Miguel Servet, 50009 Zaragoza, Spain; etambo@usj.es

**Keywords:** cultural competency, nursing education, qualitative research, transcultural nursing

## Abstract

Cultural competence is an essential component in providing effective and culturally responsive healthcare services, reducing health inequalities, challenging racism in health care and improving patient safety, satisfaction and health outcomes. It is thus reasonable that undergraduate nursing students can develop cultural competency through education and training. The aim of this paper was to investigate nursing lecturers’ perception and experience of teaching cultural competence in four undergraduate nursing programs. A phenomenological approach was selected to illicit nursing lecturers’ perception of culture and experience of teaching cultural competence. Semi-structured personal interviews were held with a sample of 24 lecturers from four European universities. The anonymized transcripts were analyzed qualitatively following Braun and Clark’s phases for thematic analysis. Six themes and fifteen subthemes emerged from thematic analysis of the transcripts. Cultural competence was not explicitly integrated in the nursing curricula. Instead, the lecturers used mainly examples and case studies to illustrate the theory. The integration of cultural content in the modules was unplanned and not based on a specific model. Nursing programs should be examined to establish how cultural content is integrated in the curricula; clear guidelines and standards for a systematic integration of cultural content in the nursing curriculum should be developed.

## 1. Introduction

According to the European Commission [1], “social inclusion is at the core of the European Social Model and European values enshrined in the Lisbon Treaty”. Nevertheless, in the past few years, social exclusion and inequality have emerged as a major concern in European society. European higher education institutions (HEI) have a responsibility to address these issues through the promotion of social, civil and transcultural competences, democratic values and fundamental rights, social inclusion, non-discriminating active citizenship and critical thinking [2]. Transcultural Nursing: A European Priority, a Professional Responsibility (TC-Nurse), is a project funded by the Erasmus+ program under Key Action 203 Strategic Partnerships for Higher Education. It represents a collaboration between four European HEI: Universidad San Jorge, Spain; Instituto Politécnico de Portalegre, Portugal; Artesis Plantijn (AP) University of Applied Sciences and Arts, Belgium; Istanbul Aydin University, Turkey. In this paper, we present the results from our investigation of European lecturers’ perception of culture and experience of teaching cultural competence in four undergraduate nursing education programs offered by the aforementioned universities.

According to Gallegos, Tindall y Gallegos [3], the concept of cultural competence first appeared in the nursing literature in the 90s. Since then, the construct of cultural competence has received a significant degree of attention [3,4] from nursing and healthcare scholars, due in part to the increased diversity of the population in developed countries [4]. The ever-growing volume of literature produced on the concept of cultural competence highlights its importance as a tool for “effective communication, intervention, and outcomes in today’s multicultural environment pervasive in the healthcare professions” [3]. However, the meaning of cultural competence is ambiguous in the literature [3,5]. According to Cai [5], there is debate on the conceptual understanding of the concept, partly due to the range of different terms used interchangeably in the literature, namely cultural competence, cultural safety, cross-cultural competence, transcultural nursing, cultural humility [6] or transnational competence [7]. Perhaps one of the most widely accepted definitions of cultural competence in the nursing field is Campinha-Bacote’s [8], who suggested that cultural competence involved understanding and appreciating similarities and differences in health beliefs and behaviours, recognising and respecting differences within and between cultural groups and being able to adapt one’s practice in order to guarantee high quality, culturally mindful, safe and effective care for all [2,8]. 

A changing society with free movement of citizens within the EU, distant parts of our globe becoming more accessible, and an increasingly multicultural population are factors influencing healthcare, highlighting the need for cultural competence and cultural awareness among healthcare professionals [9,10,11,12]. European health services designed to meet the needs of static monocultural populations are thus required to review their ability to meet the needs of diverse patients, families and communities [13]. Therefore, providing culture-specific care to the myriad culturally diverse populations has become a European priority and a professional responsibility [10,14,15]. Furthermore, the Global Standards for the Initial Education of Professional Nurses and Midwives [16] advises nursing and midwifery schools to train graduates who are able to demonstrate cultural competence.

Nursing care should address the social, cultural and biological determinants of health in order to ensure equitable health outcomes for all. Quality cross-cultural care that explicitly targets structural barriers to health such as discrimination is necessary to achieve health equity. Therefore, cross-cultural and antidiscrimination care, in addition to care targeting the social determinants of health, are expected standards of the nursing profession, and thus should be expected components of undergraduate curricula [17,18,19,20,21,22,23]. 

European HEI are becoming increasingly aware of the need to respond to diverse patient populations. Nurses are expected to be able to provide appropriate care for diverse groups, and to ensure that these patients’ human rights are respected regardless of (but not limited to) their racial, ethnic, socioeconomic, religious background and gender [12,14,15,24]. However, cultural competence is a relatively new area of concern for nurse educators in Europe and there is limited knowledge and understanding of these skills [13,25,26,27,28]. A brief scoping review of nursing curricula in Spain, Belgium, Portugal and Turkey revealed a fragmented, non-consistent approach to the concept [19,29]. For example, in Spain, only 4 out of 94 undergraduate nursing programs offered during the academic year 2019-2020 included the term transcultural, 7 included the term multicultural and 1 included the term intercultural in their program descriptions [30]. This is in agreement with Bohman and Borglin’s [31] assertion that European nursing curricula lack detailed transcultural nursing content. In fact, there is concern among nursing scholars regarding nursing students’ ability to meet the demands presented by multicultural societies and demonstrate cultural competence [32,33]. Caring for a culturally diverse patient population poses plenty of challenges [34]. European nurse educators have a dual responsibility: (1) to help students develop cultural competence by implementing creative, evidence-based educational activities that promote positive, cultural competence learning outcomes and, subsequently, (2) to contribute to enhance the quality of care provided to diverse patient populations [10,35].

### Theoretical Perspective

Transcultural nursing involves the study of cultural differences and similarities in health and illness emerging from each cultural group’s underpinning societal and organizational structures [13]. Such understanding of cultural values, beliefs and practices is the foundation for culturally congruent care; care that meets every patient’s needs, is meaningful to them and supports their lifestyle [36]. In the words of Madeleine Leininger [37], cultural competence in nursing is “defined as a formal area of study and practice focused on comparative holistic culture care, health and illness patterns of people with respect to differences and similarities in their cultural values, beliefs, and lifeways with the goal to provide culturally congruent, competent and compassionate care.” Cultural competence is thus a broad and complex construct which enables healthcare professionals to acquire “the attitudes, knowledge and skills necessary for providing quality care to diverse populations” [38] while taking into account their cultural background [32], including patients’ health and illness beliefs, religious influences, their primary language, values and other cultural factors that influence their health. It is an essential component in providing effective and culturally responsive healthcare services, reducing health inequalities, challenging racism in health care and improving patient safety, satisfaction and health outcomes [17,26,28,39]. According to several authors [24,40,41], it is reasonable that undergraduate nursing students can develop cultural competency through education and training. 

There is a growing body of evidence about nursing students’ perceptions of their own cultural competence, as well as their opinion and impact of diverse training courses on their ability to deliver culturally competent nursing care. In terms of the healthcare teaching staff’s perception and experience of integrating cultural content in the curricula, previous studies [42,43,44,45] have concluded that healthcare faculty may benefit from specific training in order to improve their knowledge of cultural issues and affectively address these issues. However, less is known about the nursing lecturers’ perception and experience of teaching cultural competence as part of undergraduate nursing curricula. Therefore, the aim of this study was to investigate nursing lecturers’ perception of culture and experience of teaching cultural competence in four undergraduate nursing programs offered at four European universities. 

## 2. Materials and Methods

### 2.1. Design

A phenomenological approach was selected to illicit the nursing lecturers’ perception of culture and experience of teaching cultural competence in four European nursing degree programs. This is appropriate as phenomenological research is a qualitative research method which seeks to understand complex phenomena through the participants’ lived experience, meaning and perspectives [46,47]. 

The COREQ reporting guidelines were used in both the framing and reporting of this study [47].

### 2.2. Setting

This article presents the results from the investigation carried out as part of the first year of the EU funded project “Transcultural Nursing: A European Priority, a Professional Responsibility. for Higher Education”. The study took place at four European universities, namely Universidad San Jorge (Spain), Instituto Politécnico de Portalegre (Portugal), AP University of Applied Sciences and Arts (Belgium) and Aydin Istanbul University (Turkey). Written permission from the participating HEI to recruit participants was sought and obtained prior to the investigation.

### 2.3. Participants

The study target population consisted of nursing educators from four European undergraduate nursing programs. We recruited a total sample of 24 nursing lecturers, seven from Spain, seven from Portugal and seven from Turkey and three from Belgium. A convenience sample representing nursing lecturers from a variety of areas of expertise was recruited according to the following inclusion criteria:

(1) Nursing lecturers employed at one of the participating HEI teaching in an undergraduate nursing study program.
(2) Lecturers who agreed to the conditions of the study and gave informed consent to participate.

### 2.4. Methods

Semi-structured, one-to-one interviews took place at the participants’ university and in the participants’ own language; interviews were conducted by an academic from each of the study sites. The interviews took place between March and August 2019 and lasted between 15 and 45 minutes. One-to-one interviews were chosen because they can elicit rich, culturally grounded insights into people’s experiences [48]. All of the data were audio recorded and transcribed verbatim. The results were later translated into English by the academic undertaking the interview, all of whom were fluent in English. All of the researchers were experienced qualitative researchers with professional health backgrounds of either a nurse or a nutritionist. All of the researchers used the same (previously agreed) interview guide. The interview questions addressed topics such as how one’s own culture. may influence one’s teaching practice; how, when and where cultural issues were addressed during lectures; whether participants had had formal or experiential training in cultural competency; and how difficulties emerging from a multicultural student group were dealt with (Table 1). 

The participants were also invited to complete a sociodemographic questionnaire with the aim of describing our sample, including variables frequently associated with a high level of cultural competency.

### 2.5. Data Analysis

Descriptive statistics was used to analyze the sociodemographic data using frequency and percentage for qualitative variables and mean and standard deviation for quantitative ones.

Two researchers, I.A.-S. and E.T.-L., analyzed the transcripts separately and derived themes and subthemes from the data. After a process of comparing and discussing the data, consensus between the researchers was reached. Six themes and fifteen subthemes emerged from thematic analysis of the transcripts. A summary of the themes and subthemes identified, illustrated by representative examples of quotes, is presented in Table 2. The codes assigned to each of the participants do not identify their university of origin in order to protect their personal identities.

The anonymized transcripts were analyzed qualitatively following Braun and Clark’s [49] phases for thematic analysis, namely (1) familiarizing with data, (2) generating initial codes, (3) searching for themes, (4) reviewing themes, (5) defining and naming themes and (6) producing the report. Quality was guaranteed by using the following techniques:

- Space triangulation was achieved by interviewing lecturers from different European HEIs to check consistency of findings over multiple sites.
- In order to enhance the rigor of the study, two researchers (I.A.-S. and E.T.-L.) analyzed the transcripts independently.
- An audit trail was kept of the researchers’ decision-making process and critical self-reflection.
- Field notes, including personal reflections on how interviews were conducted, how participants responded to the interview process and any key points arising from the interviews, were taken.
- Frequent contact between authors was maintained in order to promote further discussion on emerging themes and potential biases.

### 2.6. Ethical Considerations

Approval from San Jorge University Research Ethics Committee (study reference number 16-2019) was sought and obtained. In addition, each university gave explicit permission to collect data. Every effort was made to safeguard the participants’ anonymity and confidentiality. The lecturers participated voluntarily in the study procedures following explanation of the study which included information regarding their right to opt out at any time during the process and decline to participate in the study without any effect on their professional career. All the participants were asked to give informed consent prior to being interviewed.

## 3. Results

The sociodemographic and cultural characteristics of our sample are presented in Table 3. The average age of our participants was 40 years. Most of interviewees were female (70.8%), married (83.3%) and had a white ethnic background (95.8%). All of our participants described themselves as middle or high social class, and all of them were born in the country where they were working at the time of being interviewed. Worth mentioning is the fact that almost 80% of our participants spoke at least another language and almost 90% had prior experience of caring for patients from different cultural backgrounds. Finally, only a minority had had prior training in cultural competency and/or had lived or studied abroad for over three months (29.2% and 20.8%, respectively).

### 3.1. Theme 1. Concept of Culture

It was important, before delving into the lecturers’ experience of teaching cultural competency, to ascertain their understanding of culture.

#### 3.1.1. Subtheme 1.1. Meaning of Culture

We identified a variety of opinions and descriptions of culture. Some of our participants associated culture with race, ethnicity and nationality, whereas others described a much broader construct.

‘It is where you come from, no? People that come from a different country, also have a different culture; there are differences even within the same country, no? We have students who come from different regions, or who are from the same country, but their ethnicity is different, like being a gypsy; their culture is different’

(L6)

‘I understand culture as a space, right? Like a moment in which there are various ways to understand the same concept, in this case health’

(L5)

#### 3.1.2. Subtheme 1.2. Cultural Diversity in the Classroom

Usually, the lecturers’ understanding of culture was reflected in their perception of multiculturality in the classroom. That is, those who generally associated culture with aspects such as race and ethnicity also identified these features in their students.

‘What do you mean? Like having students from different places?’

(L1)

Whereas those who described a broader concept of culture, also tended to identify a wider variety of cultural characteristics in their audience.

‘There are people from different age groups, there are people…, this is an intuition, okay? From different educational levels, people who have other diplomas or degrees. I don’t remember having students from a different race, or culture. I do believe we once had a female student who was a gypsy’

(L2)

#### 3.1.3. Subtheme 1.3. Factors Influencing Their Concept of Culture

Our participants reflected on those factors shaping their current understanding of culture. Some highlighted specific training courses or studies, some referred to personal life experiences and many of the lecturers made reference to their previous or current experience as qualified nurses or midwives as a key influential factor.

‘Yeah, I don’t have any, um, I haven’t had any lessons about that. Just from my own experience. Both in the care sector and in the educational setting...’

(L9)

‘No, none. With that name and all that…, I am too old! Perhaps life, yes’

(L1)

‘Well (puffs), it is a bit intuitive and by experience. It is true I have been trained, especially with regard to women’s care. I think being and anthropologist also gives you a perspective, of that I am sure’

(L2)

### 3.2. Theme 2. Perception of Own Culture

Our participants reflected on their own cultural background in relation to how it influenced their teaching practice.

#### 3.2.1. Subtheme 2.1. Impact of Own Culture on Teaching Practice

The majority of our participants admitted that they had never considered whether, nor how, their own cultural background influenced their teaching practice. When they were asked to elaborate on their initial responses, they were able to identify which features were influential on their approach to cultural competency in the classroom.

‘I’ve never thought about that before., I was raised a Catholic, but I don’t believe in anything myself, so gosh, I don’t know if that really affects my lessons. I don’t think so because of the fact that I don’t believe in anything specific’

(L8)

‘Yes, I guess one is influenced by his or her own culture and beliefs consciously or sub-consciously. I try to utilize the positive aspects of my culture and family tradition in my professional life, for example, having empathy towards others, showing tolerance towards others’ beliefs and cultures, impartiality in teaching as well as clinical practice and likewise’

(L15)

#### 3.2.2. Subtheme 2.2. Culture as a Source of Misunderstanding

The lecturers described instances in which difficulties arose in the classroom, which were attributable to cultural differences between them and the students, or to specific personal opinions and characteristics. In particular, gender issues and religion were mentioned as a source of uneasiness, especially in the case of the Turkish lecturers.

‘Since my classroom has mostly Muslim students, if I had to speak on a topic that is against the norms of the religion and culture, I would find that somewhat difficult. Discussing sexuality and sex related topics is difficult for me’

(L11)

In the case of the Spanish lecturers, conflict arose more frequently between the lecturer and the students, and between the students themselves, when certain topics were presented in a different light or from a different perspective.

‘They (the students) completely misunderstood me… That was the problem, that they did not accept that there was a problem. It is in their culture to see all this as completely normal... (sight). When it really isn’t (normal). I tend to get myself into this sort of jams’

(L1)

The students’ attitude in the classroom was also identified as problematic. For example, one of the lecturers explained her frustration at certain behaviors displayed by the “foreign” students, as opposed to the more acceptable behavior demonstrated by the “local” ones.

‘Yes, they had for example a very different style of life, those typical things. They are less alert than the local students in class and that is certainly not the case with all of them. But that is very typical for the group where there are a lot of students of foreign origin, that my lessons there are a bit more difficult...’

(L10)

### 3.3. Theme 3. Teaching and Learning Cultural Competency

Our participants were responsible for delivering a wide range of undergraduate modules including, but not restricted to, Mental Health, Fundamentals of Nursing, Public Health, Children’s Care, Women’s Care and Nursing Ethics. They described the content, the teaching and learning activities and the educational methodology employed when teaching cultural competence to undergraduate student nurses.

#### 3.3.1. Subtheme 3.1. Content

Our participants identified a range of different topics addressed during lectures in relation to cultural competence. Specifically, they addressed values (e.g., empathy and respect), knowledge (e.g., in terms of features or characteristics generally attributable to a specific cultural group) and skills (e.g., communication, active listening, negotiation). Often, they described specific examples used to illustrate cultural difference, or a particular approach to care for specific individuals or groups. The following topics were mentioned more frequently by our participants: age, religion, modesty, person-centered care, end of life care, mental health care and women’s care.

‘My specialization is Principles of Nursing, and as a part of my course I do teach students how to fill up and evaluate a patient identification form. For example, they should record all information pertaining to the patients’ culture and background; preferences regarding food/nutrition or objection to treatment as blood transfusion, or organ transplantation’

(L14)

‘I talk a lot about what I call negotiation, that is, to abandon the idea that this is mine and it is all that matters, and the rest doesn’t count, no? That is not possible, and it is a lie. I think life is pure interpretation; (…) you have to negotiate and agree with whoever is in front of you. You are nobody to discard their opinions or preferences’

(L2)

#### 3.3.2. Subtheme 3.2. Teaching Methods

In terms of the methods employed in the teaching and learning of cultural competence, a range of different strategies were mentioned by our interviewees, in particular, case studies, debates, clinical simulation, tutorials and stand-up lectures.

‘I present case studies and some of them are transcultural patients, which I present for open discussion in class. We discuss the observational cases as witnessed by the students during their internships and, in this context, we also dwell on feedback provided by the foreign students in my class and I think this is quite an effective approach as far as teaching and learning activity related to cultural competence’

(L15)

‘They read a text and then there is a debate about Ulises syndrome and those things…, about how a migrant faces their new reality and all that. The problem is that they don’t participate much’

(L3)

‘In the case of prescription (module) I give a lecture, purely theoretical’

(L2)

There were instances in which the lecturers did plan to address cultural issues as part of specific sessions. However, more often than not, the integration of cultural content in their respective modules was unplanned.

‘I insert that content as I go. Often, it isn’t planned; it just happens. Circumstances, examples that kind of sneak in. I don’t plan for these things, but they happen, and quite frequently too!’

(L1)

Cultural content was generally not described in their respective module guides and, therefore, it was not assessed.

‘I don’t assess cultural content. At most, I have asked them if they think that culture should be taken into account when prescribing’

(L2)

### 3.4. Theme 4. Integrating Cultural Competence in the Nursing Curriculum

Our participants reflected on the current degree of integration of cultural content in the nursing curricula, and suggested a range of values, knowledge and skills which, in their view, should be taught to undergraduate nursing students.

#### 3.4.1. Subtheme 4.1. Values

Our interviewees mentioned a number of values and attitudes that, according to them, should be integrated into the nursing curricula. Some regretted not having learnt about them as students. Respect, justice, dignity, equity and having an open mind were often cited by the lecturers as essential to deliver excellent quality nursing care to all patients, regardless of their cultural background.

‘Tools like respect, dignity, justice and equality’

(L24)

‘Exactly, I mean, nobody taught me that when I was a student; at least I don’t remember… You know, open people’s minds’

(L6)

‘I think it’s important to know how to treat someone with respect, how to deal with that relationship appropriately and how to open it up. I think that this is a very important point, that everyone is equal and that you know how to set yourself up for this. I think they can pass that on to all students’

(L10)

#### 3.4.2. Subtheme 4.2. Knowledge

Our participants gave examples of the content that they included, or they thought should be included, in their respective modules in particular and the undergraduate curriculum in general.

‘In general, the Portuguese population has a long history of assimilation/contact with other cultures, motivated by the history of the Portuguese diaspora and by the fact that Portugal is a country with numerous former colonies in different parts of the world. Students, by analogy, have very early contact with these differences, but if these competencies are reinforced in the CLE (nursing curriculum), they will have a bigger impact’

(L18)

‘In order to be able to serve a foreign patient, nursing students must be aware of the patient’s background and culture and behave accordingly. In this respect, courses such as transcultural nursing would be very helpful for the students’

(L14)

#### 3.4.3. Subtheme 4.2. Skills

Linked to the previous elements, the lecturers mentioned a range of skills and abilities that undergraduate student nurses should develop before graduating from college. These skills were usually related to aspects such as communication, active listening and language use.

‘They should be good listeners, minute observers and excellent communicators. They should learn to accept differences in opinion and try to ease out some of their concerns of patients through proper communication and help with hospital staff’

(L16)

‘I think that communication is very important, how should you communicate with those patients, how should you…, because sometimes it is often about small things, perhaps about touching them or something like that, in certain cultures it is perhaps not so good that you touch each other in order to communicate, or perhaps in other cultures it is’

(L8)

### 3.5. Theme 5. Barriers to Teaching and Learning Cultural Competence

Our participants mentioned a number of barriers to, or difficulties in, the teaching and learning of cultural competence in the nursing curricula. We classified them into three groups, namely barriers to the integration of cultural content, barriers to the understanding of cultural content and structural barriers.

#### 3.5.1. Subtheme 5.1. Barriers to the Integration of Cultural Content

Invariably, the barriers to the integration of cultural content in the nursing curricula were related to two particular aspects, that is, time and content constraints and a perception of insufficient capacity or ability to discuss these concepts.

‘As a teacher..., (pause) I don’t have enough time to teach them everything; I don’t have time to think about what else I should add, you know’

(L4)

‘I mean, I think I have acquired the (cultural) competences through my clinical experience in the hospital, but I may not be able to pass them on to my students’

(L6)

#### 3.5.2. Subtheme 5.2. Barriers to the Understanding of Cultural Content

The lecturers complained of what they perceived as close-mindedness or perhaps rigidity of thought in their students. Occasionally, this gave way to misunderstandings and debate, which was not always conducive to learning.

‘There are certain patterns..., racism for instance. Patterns which I consider maladaptive like homophobia, racism, that we will never be able to change completely. We may perhaps be able to adjust them a little so that they (culturally diverse patients) are treated equally, but even then...’

(L4)

‘I think they have very strong, very clear opinions. Then, I don’t know if it is because their personality is already completely defined, or perhaps it is normal at their age and in the future, I don’t know, they’ll see that life is different to what you first expected. But..., there is always debate, about any topic, and I find students who are not prepared to see a reality other than the one they perceive. (...) Maybe they have learnt it at home, or from their group of friends, or the rather biased television that we have, but this is just my opinion (laughs)’

(L3)

#### 3.5.3. Subtheme 5.3. Structural Barriers

Structural barriers to teaching and learning cultural competence were mentioned by the Spanish lecturers in particular and were related to the country’s healthcare and socio-cultural contexts.

‘It always happens. When you try to explain that..., that we have to adapt to the patient and the students think that it should be the opposite, that they are here and should therefore do things as they are done here. This way of thinking should be banished if you ask me, but I am not sure that I convince them because, to do that, you need to know the reality in such a way that I don’t know if it can be explained theoretically’

(L2)

‘We have had some uncomfortable debates about..., in public health we talk about healthcare systems, and the never-ending debate about which healthcare service model is more appropriate and who should be covered by our public healthcare service. And there is always debate, like: “immigrants cannot have the same healthcare coverage as us”. But, relatively, that is also a reflection of the society we live in and, well, perhaps they say things that are politically incorrect in the classroom, but they are out there too, you know’

(L3)

### 3.6. Theme 6. Facilitators to Teaching and Learning Cultural Competence

Our participants identified aspects or characteristics, which were related to what the lecturers and/or the students knew or did in the classroom that were conducive to learning.

#### 3.6.1. Subtheme 6.1. Lecturers

The lecturers mentioned specific strategies and skills that they employed when teaching cultural competence and/or when addressing a culturally diverse audience. These were related to their language skills, to their ability to create a safe and comfortable environment in which the students felt able to express their personal opinion, and to specific strategies, such as inviting individuals from a diverse cultural background to speak about issues relating to their healthcare needs.

‘Whenever necessary, I use another language such as English, I try to identify the students’ learning needs, taking into account their experiences and trying to articulate everything with the subject’s program. In this way, I try to comply with it, meeting the diversity presented by the students themselves. In general, this strategy works when the students are proficient in Portuguese or English’

(L22)

‘While discussing the case studies, the students know that there is not “one right approach”. Everybody feels free to share their ideas or practices in their community regarding that issue. There is no bias or prejudice to any idea. This definitely creates a positive impact on the students’ learning process’

(L15)

‘Bringing someone who represents a culture; that’s what works. It would be amazing to bring a nurse who was Gypsy Roma, for example’

(L4)

#### 3.6.2. Subtheme 6.2. Students

The students, or specific groups of students, themselves were perceived as promoters. The lecturers mentioned having students from diverse cultural backgrounds both as a source of complexity and as an advantage in teaching and learning cultural competence.

‘We were talking about brain death and ictus and I remember that there was a Muslim student in the classroom; the other students kind of wanted to ask him, but they did not want to say something that could offend him, and they wanted to ask him how best to approach someone who was a Muslim and who was in that same situation’

(L5)

‘I think that was especially surprising. Uh yes, I think they can learn from each other, from each other’s culture and sometimes students don’t even think about it, because we see a lot of different cultures in our society and we are so used to that that that sometimes we don’t think about them enough, because we are different, you know what I mean?’

(L8)

## 4. Discussion

In this paper, we analyzed nursing lecturers’ perception of culture and experience of teaching cultural competence as part of four undergraduate nursing degree programs.

We asked our participants to define culture in their own words. Some of the lecturers clearly related the concept of culture to particular aspects such as race and ethnicity. This view of culture is shared by Reneau [50], who defined nursing education as “the ability to teach within the beliefs and values of students from diverse ethnic backgrounds”. However, most of them introduced additional elements or characteristics shaping their own and their students’ culture, namely age, sexual orientation, occupation and religion, among others. This is in agreement with other authors’ [41,51] view that culture is more than just race and ethnicity and that, when delivering culturally competent nursing care, nurses should be able to meet the needs of patients from different cultures on the basis of age, gender, sexual orientation, occupation, socioeconomic status, ethnicity, religious or spiritual beliefs, disability, etc. According to Aponte [52], when culture is not considered, the results may include inappropriate diagnosis, ineffective nursing interventions, lack of satisfaction and compliance and worse health outcomes. Similarly, we argue that when the teacher and the students’ culture is not taken into consideration, this may lead to conflict, misunderstanding and lost opportunities for learning. 

Both the lecturer and the students’ cultural background are influential in the process of teaching and learning; this is particularly true in the case of multicultural classrooms [2]. For example, in a study carried out in Jerusalem, Benari [53] described how Jewish Israeli and Muslim Palestinian nursing students needed to learn to understand and respect each other in order to be able to provide empathetic nursing care to their patients. In this atmosphere, nursing lecturers played a key role in dealing with tension, acknowledging difference and, more importantly, focusing on similarity rather than difference. This is a somewhat extreme example of a culturally diverse classroom. However, culturally diverse students do have diverse cultural learning needs. According to Mackay et al [54], nursing educators should be aware of, and responsive to, these needs, facilitating the students’ acculturation into both the academic and social milieu. In other words, culturally competent nursing faculty should be able to teach within, and adapt to, the cultural context of their students [55]. 

Our participants discussed how cultural differences between themselves and their students, and between the students themselves, were sometimes a cause of difficulty and conflict in the classroom. For example, one of the lecturers described the foreign students’ attitude in the classroom as “worse” than that of the local students. This may be explained by cultural differences between the foreign and the local students including their ethnicity, nationality and language. However, it may also be due to differences between the students’ educational culture, which is understood as the framework in which educational activities take place [56]. For example, some countries have a longer tradition of student-centered learning; this is the case of countries such as the UK and Belgium, whereas some other countries rely heavily on a more teacher-centered approach. It is therefore not surprising that students from diverse cultural backgrounds, who have been largely exposed to a different educational culture, behave in what can be interpreted by the teacher as an inappropriate or disinterested manner. Thus, we argue that nursing lecturers should be aware of the learning needs of diverse students and select teaching techniques that suit their specific characteristics. In fact, the need for academic and cultural support of diverse students is widely acknowledged [54]. According to Arunasalam and Burton [57] and Mackay et al [54], diverse students face three main challenges: difficulties with language if the local language is not their mother tongue, differences in education style, and social integration and connectedness. Nursing educators have an essential role to play in helping to create a positive learning environment for culturally diverse learners [58]. Specifically, Adeniram and Smith-Glasgow [58] proposed five strategies for creating and promoting a positive learning environment, that is developing nursing educators’ awareness and sensitivity, requiring education on inclusion and diversity for all nursing educators, incorporating specific elements of cultural responsiveness, fostering intergroup relations, and implementing inclusive evaluation. Failure to adequately support the needs of diverse students, including international and exchange students, results in negative student outcomes. 

None of the four nursing degree programs explicitly integrated cultural competence in the curriculum. However, the majority of our participants, with the exception of four of the Portuguese lecturers, integrated cultural content into their lectures. This usually happened on an unplanned basis and as illustrative examples of the theory and case studies, frequently emerging from the lecturers’ own clinical practice. Our participants utilized a range of teaching methods to discuss cultural content including case studies, debates, clinical simulation, tutorials and stand-up lectures. These teaching strategies have also been described in the literature. For example, out of classroom cultural immersion experiences [39,40,59,60], case studies [61], liaison and participation of service users from a diverse cultural background [14], sociocultural activities [27], class debate and discussion [18] and photovoice [62]. Similarly, none of the lecturers were aware of, nor implemented, a specific model or teaching strategy for assisting student learning about caring for patients from different cultures. This is despite the fact that models for implementing cultural content in the nursing curriculum have been developed in both the USA [17,38,63], as well as in Europe [64,65]. This apparent lack of planning, according to Bond et al [12], may explain nursing students’ relatively low level of cultural knowledge. 

The lecturers identified a number of aspects promoting the acquisition of cultural knowledge, skills and attitudes. In particular, one of the Portuguese lecturers used English as a medium of instruction when the students’ level of Portuguese was insufficient; the ability to create a safe and inclusive learning environment was also frequently mentioned as a promoter of student learning. Finally, most of the lecturers described the multicultural environment as conducive to student learning. Previous studies [50] have identified factors that promote cultural competence. These include increase nursing teachers’ level of cultural competency, validating the students’ cultural identities in the classroom and maximizing learning opportunities for their students by finding alternative ways to teach the same material.

Our participants described similar barriers to teaching and learning cultural competence in higher education. Specifically, the lecturers identified barriers to the integration of cultural content in their various modules, barriers to the understanding or acceptance of cultural content on the students’ part and structural barriers deriving from each country’s health system and social context. It was interesting to observe that all of our participants perceived cultural competence as an addition to, and not an essential component of, their respective modules. Other factors hindering the integration of cultural content in the nursing curricula included lack of teaching time and rigid syllabuses designed by third parties (e.g., accrediting bodies such as professional councils and government agencies). Interestingly, most of the Spanish lecturers also perceived the society as having a negative effect on the students’ capacity to acquire cultural competence knowledge, skills and attitudes. In their view, the Spanish students’ attitude towards cultural diversity mimicked certain prejudices and discriminating views of the general Spanish population, posing problems when certain topics were addressed in class. For example, gender issues, case studies involving Gypsy Roma patients and access to healthcare. Unfavorable outcomes in the integration of transcultural nursing in the curricula have been linked to a number of factors including: difficulty fitting it into an already full curriculum, lack of experience in this area [28,34,59] the staff’s lack of preparation and confidence to teach cultural competency concepts [14], emphasis on increasing knowledge rather than on developing culture-specific skills [12], lack of support to formally train faculty members, insufficient effort on the part of the accrediting bodies to include integration and application of transcultural nursing in the curricula [66], and a lack of consistent measurement approaches, using valid and reliable tools, which has made it difficult to interpret differences across studies [63]. This reveals a need for clear guidelines and standards for a systematic implementation of cultural awareness into the curriculum [21,34,66], as well as a thorough examination of how undergraduate nursing programs incorporate issues of culture and cultural competency in their curricula [24,63]. Based on our results, we recommend that future research in this area focuses of clarifying the barriers to the integration of cultural content in the nursing curricula and assesses the effectiveness of interventions to overcome them. These may include teacher training programs, interventions to increase the adaptability of the nursing curriculum to the needs and demands of society and specific strategies to promote cultural competence, such as the involvement of service users belonging to cultural minorities in classroom activities.

### Limitations

Although it is not usually the nature and purpose of a qualitative investigation to involve a representative sample of the target population, we wish to draw attention to the fact that only three lecturers from Belgium were recruited to participate in this study. The three Belgian lecturers did provide interesting and insightful information; however, it is possible that a larger sample of Belgian participants may have provided an additional or an alternative perspective.

## 5. Conclusions

Cultural differences between teachers and students, and between the students themselves, were sometimes a cause of difficulty and conflict in the classroom. However, learning in a multicultural environment was also perceived by the lecturers as promoting the acquisition of cultural knowledge, skills and attitudes. Barriers to the implementation of cultural content in the undergraduate nursing curricula included time and syllabus constraints; specific topics, such as gender and religious issues, which were deemed as difficult to deal with in the classroom; and deeply rooted prejudices and opinions, frequently influenced by society. Cultural competence was not explicitly integrated in the nursing curricula. Instead, the lecturers mainly used examples and case studies to illustrate the theory. The integration of cultural content in the various modules was unplanned and not based on a specific strategy or model. Nursing programs should be examined to establish how cultural content is integrated in the curricula; clear guidelines and standards for a systematic integration of cultural content in the nursing curriculum should be developed. Further research on effective teaching strategies and patient outcomes is needed in order to address and clarify these issues.

## Figures and Tables

**Table 1:** Topic guide for the semi-structured interviews.

Opening question
I am interested in hearing about your experience of teaching cultural content to nursing students. Do you address cultural issues during lectures? How and when do you actually do it?
Follow up questions
Do you usually teach in a multicultural classroom? Can you describe the various cultural groups represented in the classroom? Can you tell me more about your teaching methods/teaching and learning activities in relation to cultural competence/content? What works and what doesn’t, and why do you think that happens? What impact do you think these activities have had on the students? What aspects relating to caring for patients from diverse cultural backgrounds do you think should be taught to nursing students? Have you ever considered how your own culture influences your teaching practice? Are you confident in your level of cultural competence to teach students about caring for diverse patient populations? Have you had any formal training and/or experiential learning in cultural competence? What do you think are your training needs in this area? Under what circumstances is it difficult to discuss cultural issues with your students in the classroom? How do you deal with conflict emerging from teaching and learning in a multicultural environment?

**Table 2:** Summary of themes and subthemes and example quotes from the personal interviews.

Theme	Sub-Themes	Selected Quotes
Concept of culture	Meaning of culture	I understand culture as a space, right? Like a moment in which there are various ways to understand the same concept, in this case health. (…) Like having different points of view depending on where and how we have been raised (L5).
		It is where you come from, no? People that come from a different country, also have a different culture; there are differences even within the same country, no? We have students who come from different regions, or who are from the same country but they ethnicity is different, like being a gypsy; their culture is different, religion is another aspect (L6).
	Cultural diversity in the classroom	What do you mean? Like having students from different places? (L1).
		There are people from different age groups, there are people…, this is an intuition, okay? From different educational levels, people who have other diplomas or degrees. I don’t remember having students from a different race, or culture. I do believe we once had a female student who was gypsy Roma (L2).
Impact of own culture on teaching practice	Training, personal and clinical experience	Yeah, I don’t have any, um, I haven’t had any lessons about that. Just from my own experience. Both in the care sector and in the educational setting... (L9).
		No, none. With that name and all that…, I am too old! Perhaps life, yes (L1).
		I had no formal training, and this fact highlights the need for structured programs of multicultural training in nursing (L19).
	Perception of own culture	I’ve never thought about that before., I was raised a Catholic, but I don’t believe anything myself, so gosh, I don’t know if that really affects my lessons. I don’t think so because of the fact that I don’t believe in anything specific. And if for example you see culture as a vision on clothing, on tattoos, on.... I think that’s pretty broad out there, too, I think (laughs) (L8).
		Yes, I guess one is influenced by his or her own culture and belief’s consciously or sub-consciously. I try to utilize the positive aspects of my culture and family tradition in my professional life for e.g., having empathy towards others, showing tolerance to other beliefs and cultures, impartiality in teaching as well as clinical practice and likewise (L15).
	Culture as a source of misunderstanding	Yes, they had for example a very different style of life, those typical things. They are less alert than the local students in class and that is certainly not the case with all of them. But that is very typical for the group where there are a lot of students of foreign origin, that my lessons there are a bit more difficult... (L10).
		Since my classroom has mostly Muslim students, if I had to speak on a topic that is against the norms of the religion and culture, I would find that somewhat difficult. Discussing sexuality and sex related topics are difficult for me (L11).
Teaching and learning cultural competency	Content	I talk a lot about what I call negotiation, that is, to abandon the idea that this is mine and it is all that matters, and the rest doesn’t count, no? That is not possible, and it is a lie. I think life is pure interpretation; (…) you have to negotiate and agree with whoever is in front of you. You are nobody to discard their opinions or preferences (L2).
		Yes, I do. In the context of a multicultural approach to paediatric patients. I emphasize the preparation that nurses should have, be attentive when receiving and providing nursing care, particularly with regard to specific aspects such as religion, ethnicity, beliefs, and nutrition (L19).
	Teaching methods	I think these gender issues could lead to…, don’t you think? I mean, they could be controversial. For example, once, in clinical simulation, we gave one of the patients a more blasé attitude, a more…, you know, and one of the students actually defied him. Well, I think that these students in the end…, they have much to learn (L5).
		So, I discuss these topics in class presenting case studies. I also present these topics for open discussion in my class (L15).
		I insert that content as I go. Often, it isn’t planned; it just happens. Circumstances, examples that kind of sneak in. I don’t plan for these things, but they happen, and quite frequently too! (L1).
Integrating cultural competence in the nursing curricula	Values	You know, we try in class, we all say it…, respect. Not only towards other cultures, but also towards different political or religious opinions, or whatever (L4).
		I think empathy as a tool for interacting with people based on universal values such as respect, dignity, justice and equality (L22).
		They should not judge individuals coming from other cultures, by their own thinking and beliefs. Good communication and empathy is very important for patient satisfaction—it also plays a role in their recuperation. These aspects of nursing care should be taught to students (L13).
	Knowledge	To give a simple example; for a patient diagnosed with Cancer, how should the news be shared to the patient or his family—this would differ in every culture. So, the nursing students must be aware of the patient’s background and culture and behave accordingly (L13).
		What I was telling you about before, the more theoretical aspects, knowing the needs of people from different cultures maybe, the more basic aspects, perhaps? (L6).
	Skills	They should be good listeners, minute observers and excellent communicators. They should learn to accept differences in opinion and try to ease out some of their concerns of patients through proper communication and help with hospital staff (L16).
		I think that, when you relate to other cultures, ages, blah, blah, blah it’s complicated; you have to work with language issues and many other things that, in truth, I do not address; only as case studies, or factors to consider (L2).
Barriers to teaching and learning cultural competence	Educational (lecturers)	As a teacher..., (pause) I don’t have enough time to teach them everything; I don’t have time to think about what else I should add, you know (L4).
		I mean, I think I have acquired the (cultural) competences through my clinical experience in the hospital, but I may not be able to pass them on to my students (L6).
	Educational (students)	Somehow, we are not able to get to some of the students... I wonder if it is something that is deeply rooted within them; I don’t know if we would capable, no matter how much effort we put in, of changing certain things that they have acquired from their family perhaps, and that they accept as an absolute and immovable truth. I don’t know (L4).
		People tend to hold on to their preconceptions. (…) Then..., it’s like something visceral. But visceralism is encouraged nowadays. Visceralism is promoted so that people are less rational..., less reflexive. So, voilá, the mess is served. I say to them: “if you are visceral, they’ll do anything with you. You have to be reflexive. You have to have inner life”. Then, okay, I can be tiresome. Some people accept it very well, and some people don’t understand or don’t care (L1).
	Social	It always happens. When you try to explain that..., that we have to adapt to the patient and the students think that it should be the opposite, that they are here and should therefore do things as they are done here. This is way of thinking should be banished if you ask me, but I am not sure that I convince them because, to do that, you need to know the reality in such a way that I don’t know if it can be explained theoretically (L2).
		We have had some uncomfortable debates about..., in public health we talk about healthcare systems, and the never-ending debate about which healthcare service model is more appropriate and who should be covered by our public healthcare service. And there is always debate, like: “immigrants cannot have the same healthcare coverage as us”. But, relatively, that is also a reflection of the society we live in and, well, perhaps they say things that are politically incorrect in the classroom, but they are out there too, you know (L3).
Facilitators to teaching and learning cultural competence	Lecturers	Bringing someone who represents a culture, that’s what works. It would be amazing to bring a nurse who was Gypsy Roma, for example (L4).
		While discussing the case studies, the students know that there is not “one right approach”. Everybody feels free to share their ideas or practices in their community regarding that issue. There is no bias or prejudice to any idea. This definitely creates a positive impact on students learning process (L15).
		Whenever necessary, I mobilize another language such as English, I try to identify the students’ learning needs, taking into account their experiences and trying to articulate everything with the subject’s program. In this way I try to comply with it, meeting the diversity presented by the students themselves (L22).
	Students	We were taking about brain death and ictus and I remember that there was a Muslim student in the classroom; the other students kind of wanted to ask him, but they did not want to say something that could offend him, and they wanted to ask him how best to approach someone who was a Muslim and who was in that same situation (L5).
		I think that was especially surprising. Uh yes, I think they can learn from each other, from each other’s culture and sometimes students don’t even think about it, because we see a lot of different cultures in our society and we are so used to that that that sometimes we don’t think about them enough, because we are different, you know what I mean? (L8).
	System	Gee, I think the fact that they are already in a program where that already has a more diverse audience, that that already helps. You create a certain context within a university of applied sciences in which the multicultural aspects are already interwoven, so I think that they will be able to take that into account more, even unconsciously, than perhaps 20 years ago. What else can we do ourselves? Um... yes, the fixed framework of traditional nursing, or what should I call that, which we sometimes give, is kind of limiting (L10).

**Table 3:** Sociodemographic and cultural characteristics of the sample (*n* = 24).

Participants’ Characteristics	N (%) or Mean (SD)
	(Total *n* = 24)
Age (years)	40.25 (9.143)
Clinical work experience (years)	10.33 (8.186)
Gender	
Female	17 (70.8)
Male	7 (29.2)
Marital status	
Divorced	1 (4.2)
Married	20 (83.3)
Single	3 (12.5)
Race/ethnicity	
Asian	1 (4.2)
Caucasian/White	23 (95.8)
Residential environment	
Rural	3 (12.5)
Urban	21 (87.5)
Socio-economic level	
High social class	3 (12.5)
Middle social class	21 (87.5)
Father’s level of education	
Bachelor’s degree	6 (25)
Vocational training	8 (33.3)
Secondary education	8 (33.3)
Primary education	2 (8.3)
Mother’s level of education	
Bachelor’s degree	3 (12.5)
Vocational training	1 (4.2)
Secondary education	12 (50)
Primary education	8 (33.3)
Country of birth and work	
Belgium	3 (12.5)
Portugal	7 (29.2)
Spain	7 (29.2)
Turkey	7 (29.2)
Mother tongue	
Dutch	3 (12.5)
Portuguese	7 (29.2)
Spanish	7 (29.2)
Turkish	6 (25)
Other	1 (4.2)
Other languages spoken	
Yes	19 (79.2)
No	5 (20.8)
Belonging to a culturally diverse family or group of friends	
Yes	10 (41.7)
No	14 (58.3)
Prior experience caring for culturally diverse patients	
Yes	21 (87.5)
No	3 (12.5)
Prior training in cultural competency	
Yes	7 (29.2)
No	17 (70.8)
Lived or studied abroad for at least three months	
Yes	5 (20.8)
No	19 (79.2)

## Data Availability

The data presented in this study are available on request from the corresponding author. The data are not publicly available due to ethical restrictions.
